# Data-driven determination of zooplankton bioregions and robustness analysis

**DOI:** 10.1016/j.mex.2024.102676

**Published:** 2024-04-02

**Authors:** Patrick R. Pata, Moira Galbraith, Kelly Young, Andrew R. Margolin, R. Ian Perry, Brian P.V. Hunt

**Affiliations:** a Institute for the Oceans and Fisheries, University of British Columbia, Vancouver, B.C., Canada; b Department of Earth, Ocean and Atmospheric Sciences, University of British Columbia, Vancouver, B.C., Canada; c Institute of Ocean Sciences, Fisheries & Oceans Canada, Sidney, B.C., Canada; d Pacific Biological Station, Fisheries and Oceans Canada, Nanaimo, B.C., Canada; e Hakai Institute, Victoria, B.C., Canada

**Keywords:** Ocean partitioning, Species distributions, Data curation, Cluster analysis, Regionalization, Plankton community data, Sampling method variability, Taxonomic variability, Selection of the optimal cluster analysis method for regionalizing zooplankton community data and robustness tests

## Abstract

Identifying biogeographic regions through cluster analysis of species distribution data is a common method for partitioning ecosystems. Selecting the appropriate cluster analysis method requires a comparison of multiple algorithms. In this study, we demonstrate a data-driven process to select a method for bioregionalization based on community data and test its robustness to data variability following these steps:

• We aggregated and curated zooplankton community observations from expeditions in the Northeast Pacific.
• We determined the best bioregionalization approach by comparing nine cluster analysis methods using ten goodness of clustering indices.
• We evaluated the robustness of the bioregionalization to different sources of sampling and taxonomic variability by comparing the bioregionalization of the overall dataset with bioregionalizations of subsets of the data.

The K-means clustering of the log-chord transformed abundance was selected as the optimal method for bioregionalization of the zooplankton dataset. This clustering resulted in the emergence of four bioregions along the cross-shelf gradient: the Offshore, Deep Shelf, Nearshore, and Deep Fjord bioregions. The robustness analyses demonstrated that the bioregionalization was consistent despite variability in the spatial and temporal frequency of sampling, sampling methodology, and taxonomic coverage.

## Graphical abstract

**Figure:**
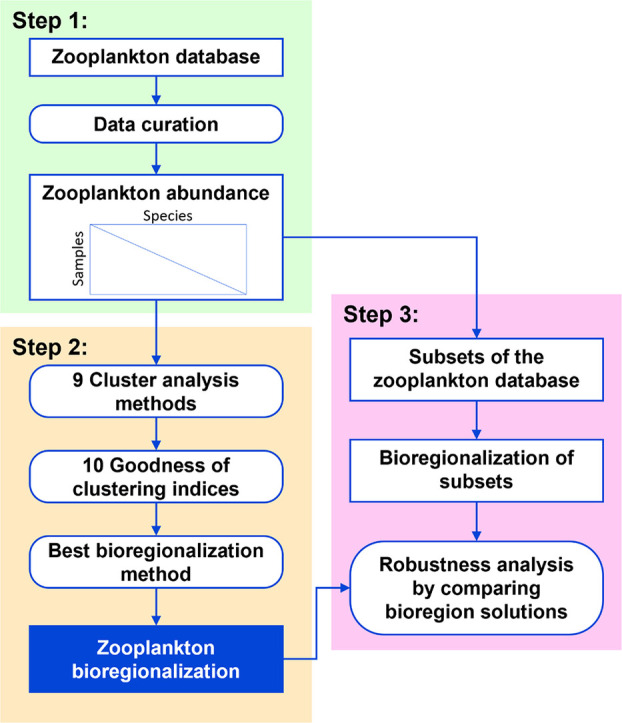

Specifications table

**Table:** 

Subject Area:	Earth and Planetary Sciences
More specific subject area:	Biological oceanography; Zooplankton ecology; Community ecology
Method name:	Selection of the optimal cluster analysis method for regionalizing zooplankton community data and robustness tests
Name and reference of original method:	R vegan package: [11]. vegan: Community Ecology Package. R package version 2.5–7. https://CRAN.R-project.org/package=vegan. R optpart package: [15]. optpart: Optimal Partitioning of Similarity Relations. R package version 3.0–3. https://CRAN.R-project.org/package=optpart.
Resource availability:	https://doi.org/10.5281/zenodo.6498999

**Method details**

## Introduction

Zooplankton have species-specific sensitivities to environmental conditions resulting in different occupied niches [2,10]. The observed distributions of zooplankton are the result of the integration of numerous environmental and biological variables experienced during their life spans. Similarities in species distributions can be used to identify biogeographic regions (bioregions) that partition the ocean into meaningful spatial units that have a characteristic community composition and oceanographic features. These units are important in systematically comparing pelagic ecosystems and can become the theoretical and practical foundation upon which other questions in biological oceanography and zooplankton ecology are explored. The increase in available zooplankton species distribution observations from large-scale monitoring programs, integrated databases, and species distribution modelling has increased the capacity for bioregionalization [4,5].

The partitioning of zooplankton bioregions is ideally achieved using the best available methodologically consistent observations at the highest possible taxonomic resolution. Aggregating historical zooplankton observation data from various sources will contain a variety of zooplankton sample collection methodologies and different taxonomic foci. The frequency of sampling may also be spatially and temporally uneven. Despite these sources of variability, synoptic analyses of zooplankton distributions are needed. After careful and purposeful curation of aggregated datasets, the important patterns of spatial biogeographic similarity may still be apparent as demonstrated by recent zooplankton bioregionalization studies [1,3,7]. Cluster analysis is a common method for determining bioregions but there is no *a priori* justification for the choice of cluster analysis method in bioregionalization studies and a comparison of multiple algorithms is recommended [8].

In this study, we aggregated and curated zooplankton community observations collected across 20 years of research expeditions in the Northeast Pacific Ocean to identify zooplankton bioregions. Our objective was to determine the best bioregionalization approach for the zooplankton dataset and to test how robust the bioregionalization results were to different sources of sampling and taxonomic variability.

## Data curation

**Fig. 1:**
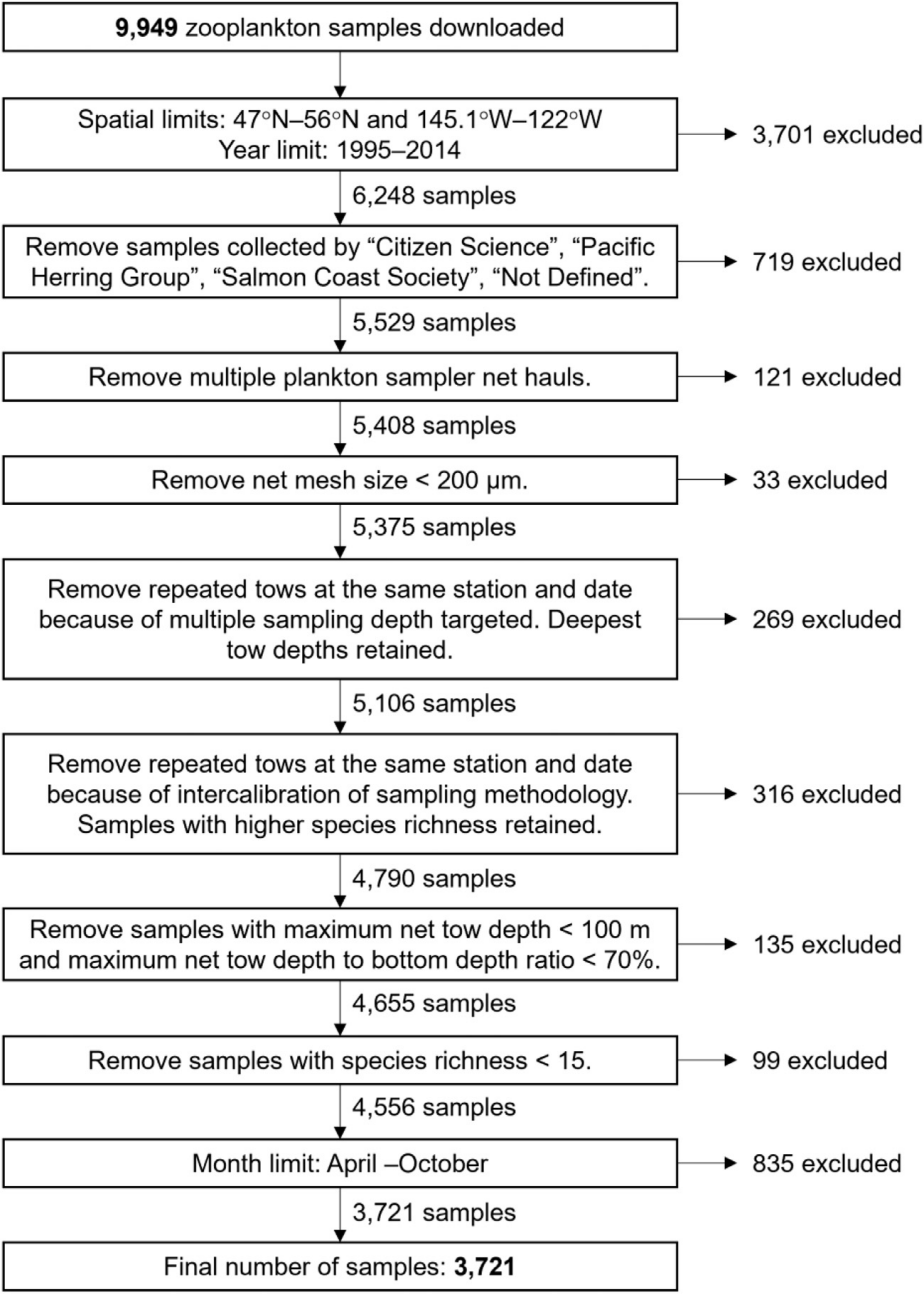
Flow diagram of the zooplankton database curation.

The zooplankton data were sourced from the *Fisheries and Oceans Canada, Institute of Ocean Science Zooplankton Database* and downloaded in July 2019. Zooplankton samples that were collected within the Canadian Northeast Pacific area with a maximum tow depth of 400 m were extracted resulting in 9949 samples. The analysis was bounded by the spatial extent of 47°N–56°N and 145.1°W–122°W and temporal extent of April–October 1995–2014. This temporal period was selected because it included the years with the most consistent taxonomic information, which limited the between-taxonomist variability in microscopic identification. The months representing the spring to summer or early fall were periods of interest in the companion paper [12] and were also the months which had the highest number of samples. The zooplankton samples were collected from various cruises and included long-running monitoring programs as well as infrequently sampled or ad-hoc stations. Various curation filters were applied to reduce the methodological heterogeneity in sampling. A minimum species richness of 15 species per sample was set to reduce the noise in the dataset, which would otherwise cluster out as outlier samples [13]. A flow diagram detailing the data curation is provided in Fig. 1.

The zooplankton dataset included 867 taxa names with observations identified at various taxonomic levels. When possible, the species-level names were retained. Taxa which were mostly identified at a coarser taxonomic resolution, despite having some records at the species-level, were assigned with the coarser level name. A flagging categorization was applied to the taxonomy to identify which records were included in the analysis and is as follows:

a. Flag = 1 Include: Either species-level or coarser-level taxonomic names.
b. Flag = 2 Exclude: Early life stages of copepods (C1-C3) identified at species level.
c. Flag = 3 Exclude: Records not identified to species level when majority of species within the taxonomic group were identified at species level.
d. Flag = 4 Exclude: Unicellular plankton from groups Ciliophora, Protista, Protozoa, and Pyrrophycophyta. Metazoans which were not sampled well in zooplankton nets, parasitic, or rarely found in epipelagic oceanic plankton samples in groups Insecta, Isopoda (except *Munnopsis*), Nemata, Nemertea, Phoronida, Platyhelminthes, Rotifera, Scyphozoa, and Sipuncula were excluded. Early life stages with unidentified taxonomic groups such as eggs, nauplii, protozoea, zoea, medusae, bracts, gas floats, etc. were excluded.

**Fig. 2:**
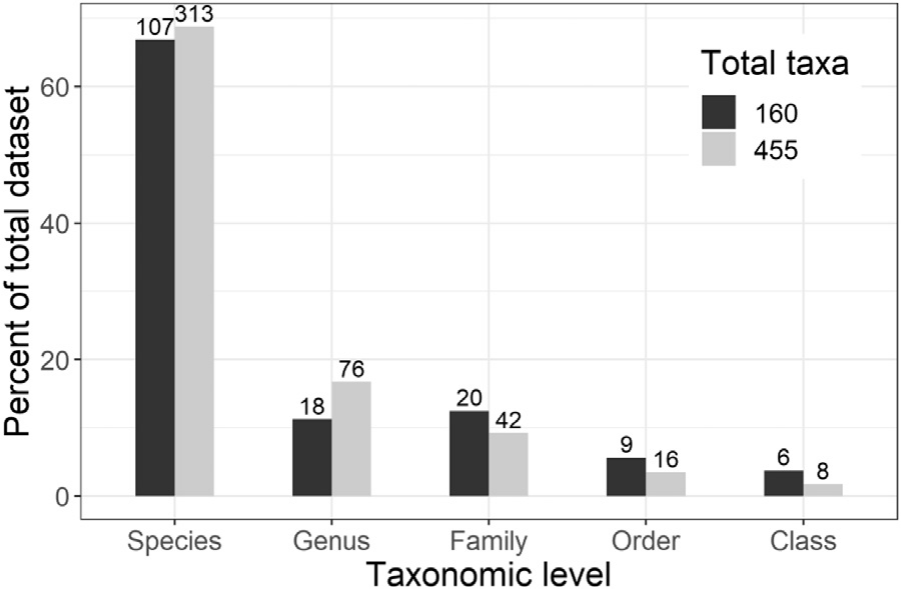
Distribution of taxonomic levels of the taxa analyzed for the complete set of 455 taxa and the 160 taxa present in ≥ 3 % of samples. The number of taxa is indicated by the value on top of the bars.

Organisms belonging to Flag = 1 totaled to 466 taxa but only 455 taxa were retained for the analysis restricted to April-October samples. Most of the taxa were analyzed at the species level (Fig. 2). Decapods were analyzed mostly at the family level, except the genus *Cancer*, while ichthyoplankton were analyzed at the order level. Class level taxa were all meroplanktonic.

## Bioregionalization method selection

The combinations of three dissimilarity matrices and three cluster analysis methods were compared resulting in nine approaches to bioregionalization. The dissimilarity matrices included the Bray-Curtis dissimilarity (BCD) of the log-transformed abundance, which is commonly used with a hierarchical cluster analysis to identify clusters based on species composition [9]. We also explored the Sorensen dissimilarity, which is the presence-absence analogue of the BCD, and the log-chord dissimilarity, which is the Euclidean dissimilarity applied to the log-chord transformed species abundances [9]. These dissimilarity matrices were calculated using the ‘*vegan’* package in R [11] and compared using a Mantel test. The cluster analysis methods included the hierarchical cluster analysis using the Ward distance, the K-means algorithm, and the Partitioning Around Medoids (PAM) algorithm. Solutions of the nine approaches were derived at *K* = 2–20 clusters resulting in 1710 clustering solutions.

Ten goodness of clustering indices from the R ‘*optpart*’ [15] and ‘*fpc*’ [6] packages were used to evaluate the cluster analysis results (Table 1). Eight of the ten indices were identified to be biologically and ecologically meaningful for community datasets [14] and two more indices were included because they were common cluster analysis evaluation indices. Half of the indices used the abundance table to evaluate the clustering solutions while the other half use the dissimilarity matrix. The log-transformed abundance table and the log-chord dissimilarity matrices were used for the evaluation in all cluster analysis methods. For each number of clusters (K), the nine clustering approaches were ranked and the median ranking across all Ks were calculated for every index. The clustering approaches were compared according to the distribution of median index rankings. The Kruskal-Wallis *H* test and the *post hoc* Dunn's test and pairwise Wilcoxon rank sum tests, both with a Benjamini-Hochberg (BH) adjustment for multiple comparisons, were used to test significant differences in cluster analysis solutions. To further explore the differences between the best approach and the standard approach of using a hierarchical cluster analysis of the BCD, the cluster analysis dendrogram was inspected and compared to the best clustering solution. The goodness of clustering indices were also used to identify the optimal number of clusters for the bioregionalization.

**Table 1:** Goodness of clustering indices used in comparing bioregionalization approaches.

Index	Basis of evaluation	Direction of ranking	R function and package
Total indicator value (Indval)	Transformed abundance table	Higher better	indval() in ‘optpart’
Indicator species analysis to minimize intermediate constancy (ISAMIC)	Transformed abundance table	Higher better	isamic() in ‘optpart’
Optimal classification using species-to-cluster fidelity (OptimClass)	Transformed abundance table	Higher better	fisher.test() in ‘optpart’
Table deviance (TABDEV)	Transformed abundance table	Lower better	tabdev() in ‘optpart’
Total chi-square interia summed across clusters (TOTCHI)	Transformed abundance table	Lower better	totchi() in ‘optpart’
Dissimilarity diameter (DISDIAM)	Dissimilarity matrix	Lower better	disdiam() in ‘optpart’
Partition Analysis (PARTANA)	Dissimilarity matrix	Higher better	partana() in ‘optpart’
Average silhouette width (Silhouette)	Dissimilarity matrix	Higher better	gensilwidth() in ‘optpart’
Within-cluster sum of squares	Dissimilarity matrix	Lower better	cluster.stats() in ‘fpc’
Calinski-Harabasz criterion	Dissimilarity matrix	Higher better	cluster.stats() in ‘fpc’

**Fig. 3:**
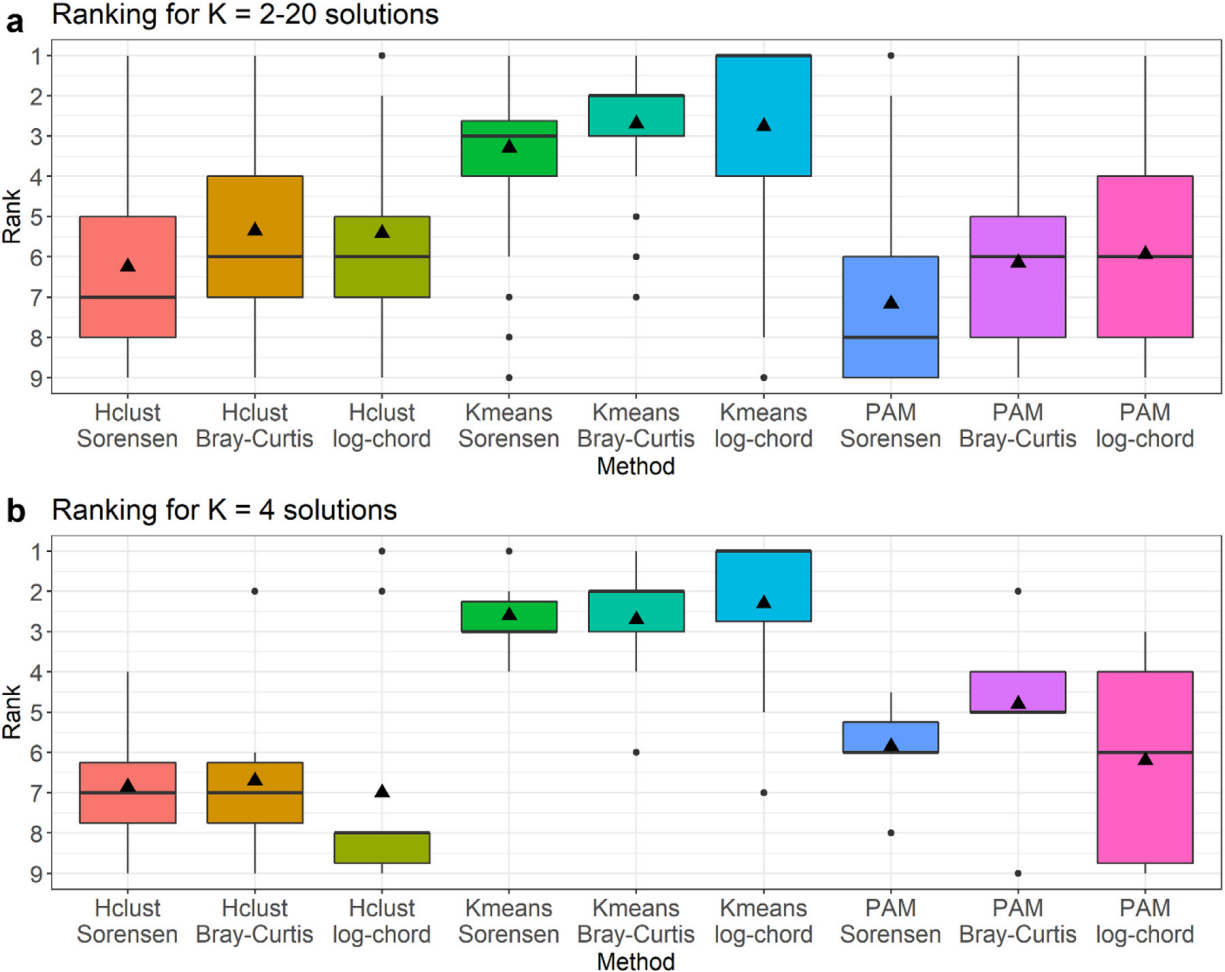
Comparison of cluster analysis methods based on 10 clustering indices for (a) *K* = 2–20 solutions and (b) *K* = 4 solutions only. Ranking closest to 1 indicates the best approach. The boxes represent the first and third quartiles, the horizontal bars represent the median, while the triangles represent the mean ranking.

A Mantel test using Pearson correlation with 999 permutations indicated that the three dissimilarity matrices were highly correlated (*r* = 0.91–0.97, *p* < 0.05). The Kruskal-Wallis *H* test determined that the cluster analysis methods were significantly different (χ^2^ = 626.4, df = 8, *p* < 0.001). The clustering method with the highest median ranking was the K-means clustering of the log-chord transformed abundance when inspecting the range of *K* = 2–20 cluster solutions (Fig. 3a). According to a *post hoc* Dunn's test, the K-means clustering of the log-chord transformed abundance was not significantly different to the K-means clustering of the BCD (*p* = 0.412) but was significantly different to the K-means clustering of the Sorensen dissimilarity (*p* = 0.025). A pairwise comparison using the Wilcoxon rank sum test identified significant differences between the K-means clustering of the log-chord transformed abundance with the K-means clustering of the BCD (*p* = 0.005) and the K-means clustering of the Sorensen dissimilarity (*p* < 0.001). For both *post hoc* tests, all the K-means clustering results were significantly different to any of the hierarchical clustering or PAM methods. The hierarchical and PAM clustering of the Sorensen dissimilarity had the lowest median rankings (Fig. 3a). On average, the hierarchical clustering of the BCD and log-chord transformed abundances similarly ranked as low as the PAM counterparts.

**Fig. 4:**
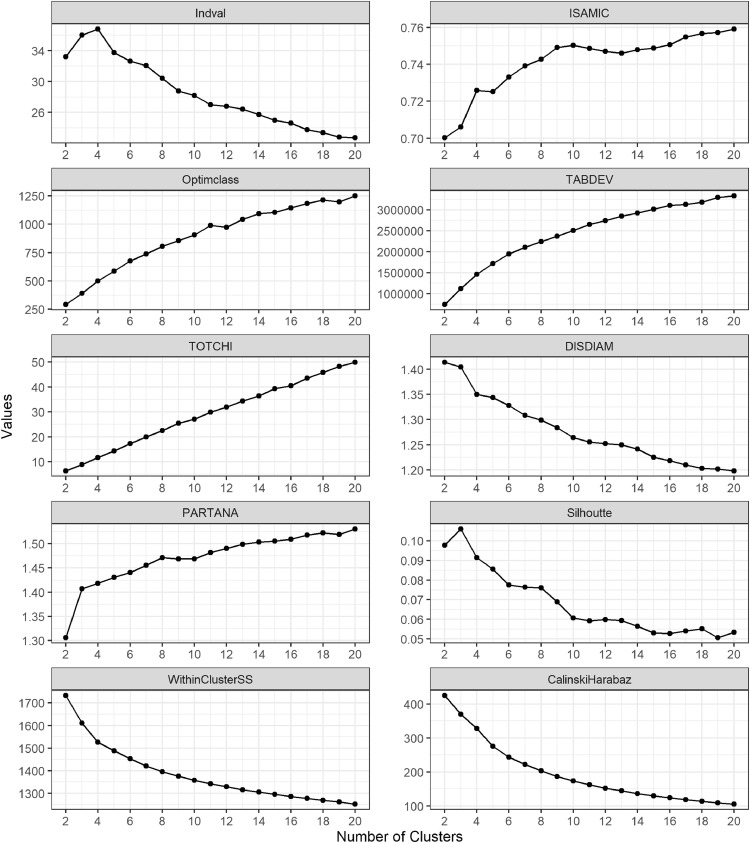
Values of the goodness of clustering indices labelled in each subplot for the K-means cluster analysis of the log-chord transformed abundance table.

In determining the best number of clusters, the highest value for the Indval and Silhouette indices were considered optimal. The elbow method was used for the other indices, in which the best number of clusters was selected when the change in index value became small. Most of the indices (Indval, ISAMIC, DISDIAM, TABDEV, and Within-cluster sum of squares) identified 4 as the best number of clusters (Fig. 4). The PARTANA and Silhouette indices selected 3 clusters and the Calinski-Harabasz criterion selected 5 clusters. The elbow was unclear for the OptimClass and TOTCHI indices and were not considered further. Ultimately, we selected *K* = 4 as the optimal number of clusters.

**Fig. 5:**
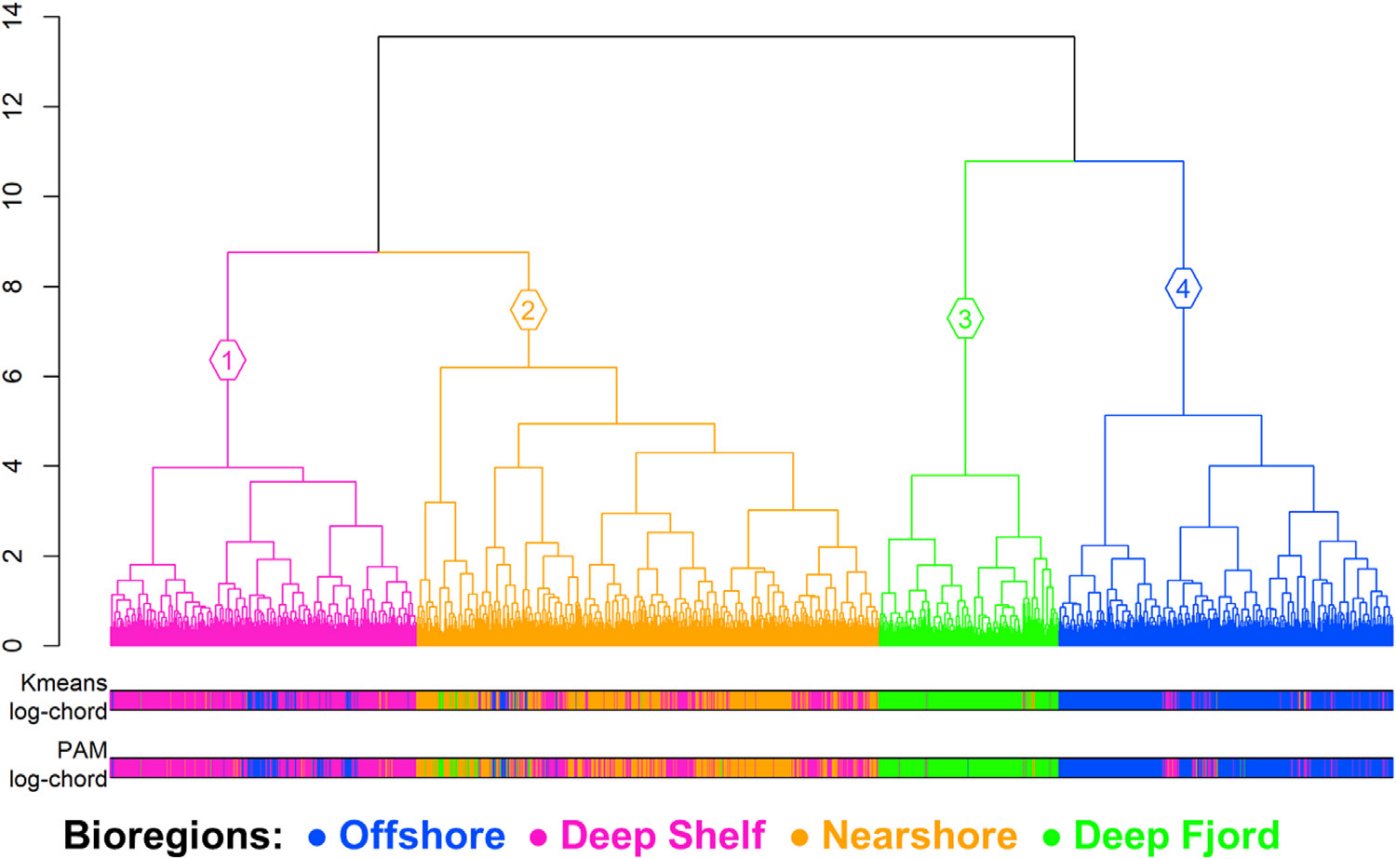
Comparison between the hierarchical clustering of the Bray-Curtis dissimilarity of log-transformed zooplankton abundance (dendrogram) versus the K-means and partitioning around medoids (PAM) clustering of the log-chord transformed zooplankton abundance.

**Fig. 6:**
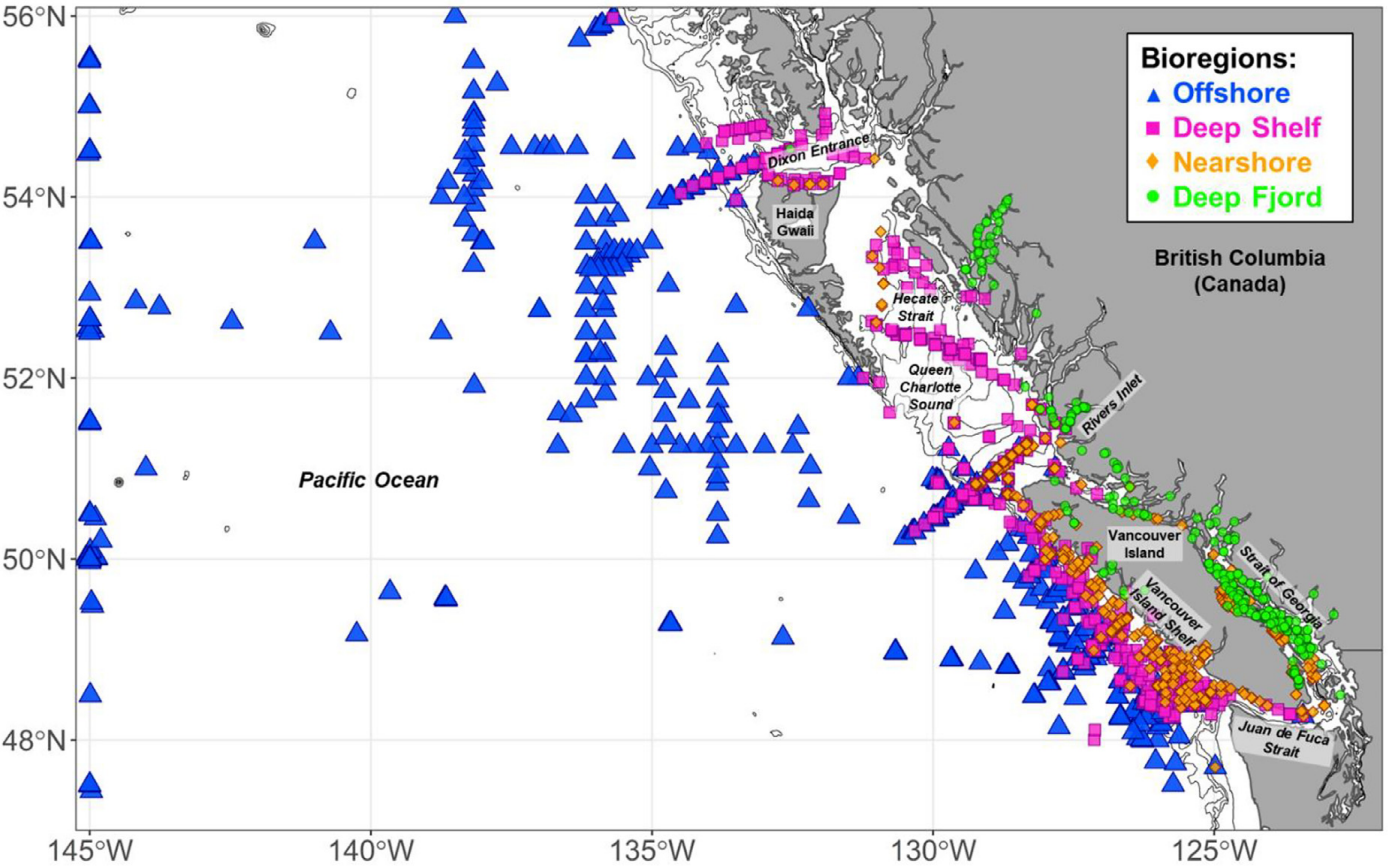
Zooplankton bioregionalization based on the K-means cluster analysis. The gray contours follow the 100 m, 200 m, 500 m, and 1000 m bottom depth contours. This figure is replicated in the companion paper [12].

We then tested to determine if the K-means clustering of the log-chord transformed abundance was the best bioregionalization approach by comparing the nine cluster analysis methods at *K* = 4 clusters only (Fig. 3b). At *K* = 4 clusters, the K-means clustering of the log-chord transformed abundance had the highest median ranking. The hierarchical, K-means, and PAM methods generally agreed with the clustering of the Deep Fjord bioregion at *K* = 4 but had multiple instances of disagreement when partitioning along the offshore to nearshore gradient (Fig. 5). The skill in partitioning the shelf is important as it is the area with the highest number of zooplankton samples in the dataset and is also the most regularly sampled. Although the standard hierarchical clustering approach confers the advantage of quantifying and visualizing the connectivity between samples through a dendrogram, its relatively poor partitioning of the shelf should not be overlooked. Thus, the K-means clustering of the log-chord transformed abundance at *K* = 4 clusters was determined as the optimal bioregionalization method for this zooplankton dataset. This corresponded to the bioregionalization of the Offshore, Deep Shelf, Nearshore, and Deep Fjord bioregions (Fig. 6). The physical and biological characteristics of these clusters are further described in the companion paper [12].

## Robustness analysis

Quantifying and understanding the effect of the sampling variability in the zooplankton data to the cluster analysis solutions are important in assessing the robustness of the bioregionalization. In this section, we calculated the classification error relative to the bioregionalization using the K-means cluster analysis of the log-chord transformed abundance at *K* = 4 clusters as determined in Section 3. Subsets of the zooplankton dataset were log-chord transformed and clustered with a K-means cluster analysis at *K* = 4. Various subsampling experiments on the number of samples and species, sampling methodology, sampling period range, spatial coverage, and taxonomic specificity and extent were tested. The bioregionalization of the data subsets were compared to the bioregionalization of the complete dataset, which was regarded as the “true” clustering solution. The percentage of samples shared by the two datasets that had a different bioregion assignment was considered as the overall classification error. The classification error was then disaggregated to determine which bioregion contributed the most to the overall classification error. The classification error is qualitatively referred here as very low (0–5 %), low (6–10 %), moderately low (11–15 %), moderately high (16–33 %), and high (34–100 %).

### Experiment 1: number of samples and species

For experiment 1 on the number of samples and species, the zooplankton dataset of 3721 samples and 160 species was randomly subsampled by reducing the number of samples or the number of species analyzed in the bioregionalization. The subsampling was done without replacement at 5 % intervals for 1000 iterations.

**Fig. 7:**
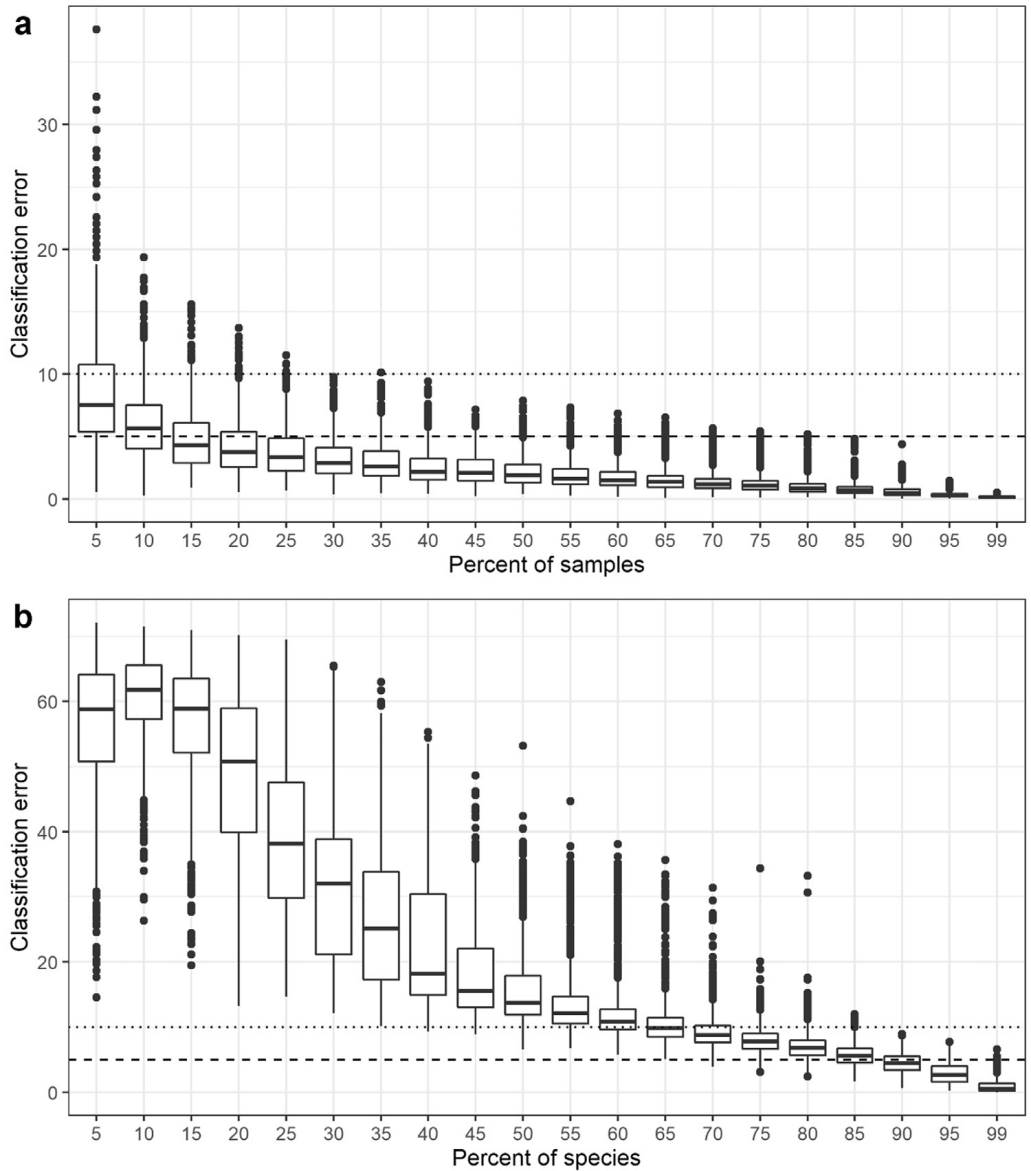
Robustness analysis on the number of (a) samples and (b) species included in the bioregionalization. The boxes represent the median ranking and the first and third quartiles and the horizontal bars represent the median. The dashed lines mark the 5 % and 10 % classification errors.

In this test, the overall range and median classification errors demonstrated that the bioregionalization was robust to the number of samples analyzed (Fig. 7a). Most of the classification errors were low, even when only 5 % of the samples were regionalized and the median error was low for at least 15 % of the samples (Fig. 7a). The consistency of the spatial differentiation to the number of samples provided confidence that the statistical analyses that utilized 89 % of the dataset due to availability of environmental data in the Pata et al. [12] companion paper represented the bioregional differentiation of the overall data. When varying the taxonomic coverage of the data, the bioregionalization was robust to a small reduction in the number of species analyzed with low errors at 65 % of the species and very low errors at 90 % of the species (Fig. 7b). This suggests that potentially not including or detecting a relatively common species in a sample does not change the overall results. Classification errors were high when less than 50 % of the species were analyzed (Fig. 7b) which is not surprising given that the differentiation of the samples was dependent on species distributions.

### Experiment 2: sampling methodology

For experiment 2 on the sampling methodology, the classification error was first determined for each category of the sampling methodology. The sampling methodology varied according to the time of the day of sampling, type of net tow, net mouth diameter, and net mesh size. The classification error was first determined for each category in the sampling methodology. For the type of net tow, net mouth diameter, and net mesh size, most samples were collected using two or three of the categories and subsampling utilizing only the dominant categories were performed. Finally, random subsampling without replacement for 1000 iterations was done by maintaining the same number of samples from each category according to the minimum number of samples in a category.

The bioregionalization was generally robust to the heterogeneity in sampling methodology (Table 2). The moderately low to high errors for some categories (Table 2) are likely explained by the low sample size of those subsets and the systematic bias in the use of a sampling method in a region (e.g., ring net vertical net haul used only in nearshore and fjord sampling or 0.56 m net mouth diameter not as commonly used in the Deep Fjord than the other bioregions). If the representation of the categories were standardized, the mean classification error was low for the sampling time (5.40 % classification error) but moderately high for the other sampling method types because of the low total sample size (20.12 %–26.22 % classification error). When only the range of dominant categorizes was analyzed, the classification errors were very low (4.42 %–4.46 % classification error). For the sampling time, most of the uncertainty occurred between the Offshore and Deep Shelf bioregions where the detection of vertically migrating zooplankton such as copepods and euphausiids may be limited during the daytime (Table 2). For the net type, mouth diameter, and mesh size, most of the uncertainty for the analyses of the dominant categories was from the Deep Shelf bioregion.

**Table 2:** Robustness analysis on sampling method heterogeneity. Instances when random subsampling was done for 1000 iterations are marked by a *. For random subsampling, the values show the mean and the range of the classification error. VNH: vertical net haul; ONH: oblique net haul.

Robustness test	N samples	Overall classification error (%)	Contribution of bioregion to overall classification error (%)			
			Offshore	Deep Shelf	Nearshore	Deep Fjord
**Sampling time**						
Daylight samples	2948	3.05	0.03	0.81	2.07	0.14
Night samples	773	9.18	4.01	5.05	0	0.13
Same number of day and night samples*	1546	5.40 (1.36–9.70)	1.84 (0–4.85)	3.38 (0.39–5.24)	0.10 (0–0.91)	0.08 (0–0.71)
**Net type**						
SCOR VNH	201	14.93	1.49	0	4.48	8.96
Bongo VNH	3166	4.04	0.79	3.19	0.06	0
Bongo ONH	260	18.46	0	18.08	0.39	0
NorPac VNH	59	38.98	0	0	30.51	8.48
Ring VNH	35	60.0	0	0	51.43	8.57
Excluding Bongo VNH	555	23.06	0	7.21	10.09	5.77
SCOR and Bongo	3627	4.44	1.16	3.12	0.17	0
Same number of net type samples*	175	21.24 (12.57–34.86)	0.99 (0–9.14)	2.85 (0–12.57)	8.73 (2.86–21.14)	8.68 (5.14–12.57)
**Net mouth diameter**						
Net mouth 0.42 m	59	38.98	0	0	30.51	0.13
Net mouth 0.50 m	136	40.44	0	0.74	31.62	8.09
Net mouth 0.56 m	2322	16.88	8.31	8.36	0.09	0.13
Net mouth 0.58 m	1204	15.20	0.08	7.48	7.48	0.17
Net mouth 0.56–0.58 m	3526	4.42	1.16	3.04	0.20	0.03
Same number of net mouth diameter samples*	236	26.22 (13.98–38.14)	0.14 (0–5.08)	4.39 (0–12.71)	16.86 (8.90–24.15)	4.83 (2.54–8.90)
**Mesh size**						
Mesh size 200–202 µm	48	52.08	0	0	33.33	18.75
Mesh size 236 µm	2345	14.20	7.38	6.65	0.09	0.09
Mesh size 250–253 µm	1262	18.86	3.88	9.19	5.71	0.08
Mesh size 330–335 µm	66	31.82	0	0	30.30	1.52
Mesh size 236–253 µm	3607	4.46	1.11	3.16	0.17	0.03
Same number of mesh size samples*	192	20.12 (10.94–42.71)	1.73 (0–9.38)	0.96 (0–10.94)	10.10 (3.65–19.79)	7.33 (5.73–9.90)

### Experiment 3: sampling period

For experiment 3 on the sampling period, a bioregionalization was derived for each month or year. The seasonal extent was subsampled for spring samples only (April–June) and for summer to early fall (July–October). The annual extent was subsampled for 5-year and 10-year intervals. Similar to the sampling methodology tests, random subsampling that maintained the number of samples from each month (170 samples/month) or year (78 samples/year) was done without replacement for 1000 iterations.

**Fig. 8:**
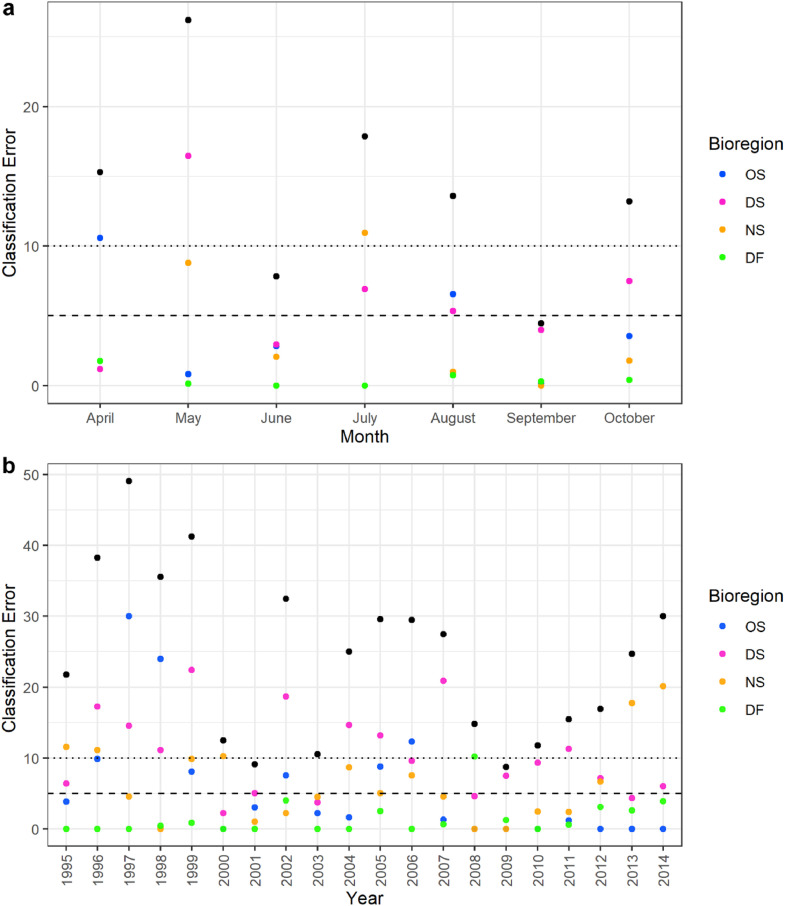
Robustness analysis on applying the bioregionalization for specific (a) months or (b) years. Black circles mark the overall classification error and colored circles are the contribution of each bioregion to this error. OS: Offshore, DS: Deep Shelf, NS: Nearshore, DF: Deep Fjord bioregions.

Deriving the spatial differentiation with data from a specific month or year had classification errors which ranged from very low to high (Fig. 8). Errors were lowest for June and September which had the greatest number of samples but the years with the lowest errors did not correspond to the years with the most samples (Fig. 8). When only the April–June samples and the first and last five years of the dataset were analyzed, the classification errors were moderately high (16.87 %–17.30 % classification error) but for the July–October samples and the other 5-year and 10-year ranges¸ the classification errors were very low to moderately low (1.63 %–10.83 % classification error) (Table 3). These suggest that the bioregionalization was dependent on capturing the temporal extent of the zooplankton communities which can be explained by how many of the species are present or have peak abundances during specific months or years. On the other hand, the bioregionalization was not sensitive to equally representing months or years, with very low average classification errors (3.27 %–3.64 % classification error) when randomly subsampling for a standardized number of samples. Classification errors in the temporal tests were lowest for the Deep Fjord bioregion and tended to be highest for the Deep Shelf bioregion (Table 3).

**Table 3:** Robustness analysis on sampling period analyzed. Instances when random subsampling was done for 1000 iterations are marked by a *. For random subsampling, the values show the mean and the range of the classification error.

Robustness test	N samples	Overall classification error (%)	Contribution of bioregion to overall classification error (%)			
			Offshore	Deep Shelf	Nearshore	Deep Fjord
**Seasonal extent**						
April–June	1678	16.87	0.54	4.59	10.13	1.61
July–October	1535	1.63	0.20	0.98	0.26	0.20
Equal number of samples from each month*	1190	3.64 (0.59–14.29)	0.96 (0–6.89)	1.47 (0–6.64)	1.04 (0–5.97)	0.17 (0–1.77)
**Annual extent**						
1995–1999	734	17.30	13.08	3.95	0	0.27
2000–2004	963	5.30	2.60	1.97	0.62	0.10
2005–2009	914	10.83	0.77	8.43	1.42	0.22
2010–2014	1110	17.03	0	3.60	11.26	2.16
1995–2004	1697	7.60	4.54	2.53	0.18	0.35
2005–2014	2024	3.71	0	1.53	1.93	0.25
Equal number of samples from each year*	1560	3.27 (0.77–11.22)	0.88 (0–5.90)	1.33 (0–6.09)	0.91 (0–5.39)	0.15 (0–1.28)

### Experiment 4: spatial resolution

**Fig. 9:**
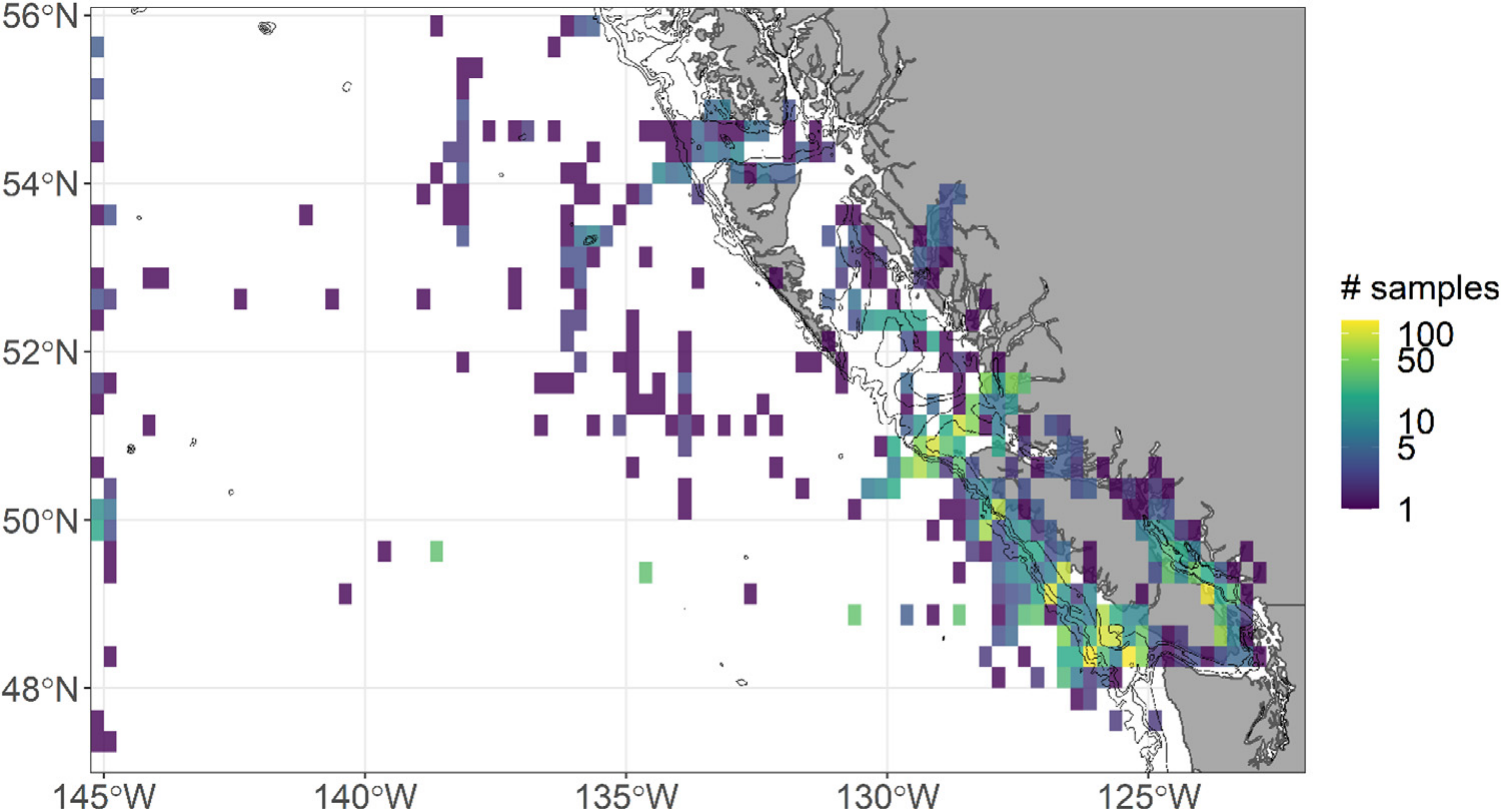
Total number of samples analyzed when gridded at a 0.25° resolution. The maximum number of samples in a grid cell is 142.

For experiment 4 on the spatial resolution, the effect of the unevenness in the spatial coverage of the samples (Fig. 9) was tested by downscaling the spatial resolution of the analysis from point locations to grid cells. The resolutions inspected were 0.01°, 0.05°, 0.10°, 0.25°, 0.50°, 1.0°, and 2.0° Random selection of samples within each grid cell was done without replacement for 1000 iterations.

The average classification errors when downscaling the spatial resolution to 0.01°–0.50° were low (Table 4). This suggests the bioregionalization was robust to the spatial autocorrelation at fine scale resolutions. Because all the samples which fell into the same grid cell were binned in the downscaling tests, grid cells composed of regularly sampled research stations were similarly represented as grid cells composed of seldom sampled areas. Thus, the results suggest that the regionalization was robust to the bias in combining these two types of sampling data. At the coarse resolutions of 1.0° and 2.0°, which are common grid sizes in regional and global analysis, the classification errors were moderately low to moderately high (Table 4). This indicates that averaging community data at coarse spatial resolutions may not capture coastal gradient well. For all resolutions, the Deep Fjord bioregion had very low classification errors and most of the uncertainty was between the Offshore, Deep Shelf, and Nearshore bioregions (Table 4). This was likely due to the gridding not resolving the depth gradient along the continental shelf and slope.

**Table 4:** Robustness analysis on spatial resolution. The samples were downscaled to different spatial resolutions and grid cells were represented evenly. Random subsampling was done for 1000 iterations. The values show the mean and the range of% classification error.

Robustness test	N samples	Overall classification error (%)	Contribution of bioregion to overall classification error (%)			
			Offshore	Deep Shelf	Nearshore	Deep Fjord
0.01° grid	1488	3.44 (0.54–13.24)	0.95 (0–6.59)	1.41 (0–5.65)	0.94 (0–5.65)	0.14 (0–1.21)
0.05° grid	948	4.10 (0.63–12.13)	1.03 (0–6.54)	1.67 (0–8.23)	1.21 (0–6.86)	0.19 (0–1.79)
0.10° grid	742	4.64 (0.54–14.15)	1.19 (0–7.82)	1.84 (0–7.28)	1.43 (0–8.63)	0.19 (0–2.16)
0.25° grid	399	6.21 (1.0–19.80)	1.53 (0–11.03)	2.54 (0–10.78)	1.88 (0–11.53)	0.26 (0–2.76)
0.50° grid	215	7.92 (0–31.63)	1.75 (0–14.42)	3.30 (0–17.67)	2.56 (0–15.81)	0.31 (0–4.19)
1.0° grid	99	12.30 (0–40.40)	2.55 (0–19.19)	5.08 (0–25.25)	4.25 (0–18.18)	0.42 (0–5.05)
2.0° grid	41	17.90 (0–46.34)	4.12 (0–21.95)	7.40 (0–29.27)	5.62 (0–24.39)	0.76 (0–12.20)

### Experiment 5: taxonomic specificity

For experiment 5 on taxonomic specificity, the taxonomic resolution was evaluated by coarsening the resolution of the complete number of samples. The dataset was analyzed at the genus and family level by aggregating the abundance of all species belonging to a genus or family. During the data curation processes (Section 2), some records were excluded because they were not identified to species level (exclusion flags 2 or 3). The genus and family level analyses were also done with the previously excluded samples. The taxonomic extent was evaluated by selecting specific zooplankton groups. Furthermore, the bioregionalization was derived without applying the filter of removing species found in less than 3 % of the samples for the complete species-level dataset and for specific zooplankton groups. Finally, the bioregionalization using the abundance data was compared to different community data types using the presence-absence and log-chord transformed biomass data.

In coarsening the taxonomic resolution of the analysis, information on the differentiation in distributions of related species in a genus or family were lost. Nonetheless, varying the minimum taxonomic level resulted in low classification errors (Table 5). This suggests that the bioregionalization would not have significantly changed due to cases for which resolving the species-level taxonomy of a group was challenging or cases in which species represented species-complexes that vary in geographic ranges. When the observations that were excluded in the taxonomy curation were included in the analyses, the increase in classification error was still low (Table 5). Limiting the analysis to specific zooplankton groups had mixed results (Table 5). Most of the species in the dataset were holoplanktonic (78 % of species) and limiting the analysis to just holoplankton had low classification error (6.61 % classification error). When only crustacean holoplankton (53 % of species) were analyzed, the classification error was moderately low (11.18 % classification error) indicating that the bioregionalization was dependent on the distribution of crustaceans which often dominate the zooplankton communities. The bioregionalization of meroplankton or of non-crustacean holoplankton was insufficient to spatially differentiate the samples. Despite dominating the taxonomic list, bioregionalization of copepods only resulted in moderately high classification error (17.20 % classification error). This suggests that spatial partitioning based exclusively on copepods may be insufficient in representing the entire zooplankton community especially in resolving the Deep Shelf and Nearshore environments. Interestingly, bioregionalization of holoplankton that are not copepods had a similar classification error (16.34 % classification error) indicating that non-copepod zooplankton are as important as copepods in defining bioregions. Including the more rarely observed species found in < 3 % of the samples did not affect the bioregionalization (Table 5). Thus, excluding the 295 species from the analysis in the companion paper [12] to reduce the noise in the data and the computation time for many of the statistical tests is justifiable. Finally, the classification errors were low when the alternative community data types were used to derive the bioregionalization although the error was lower for the presence-absence data compared to the biomass data (Table 5).

**Table 5:** Robustness analysis on taxonomic specificity and extent.

Robustness test	N taxa/ species	Overall classification error (%)	Contribution of bioregion to overall classification error (%)			
			Offshore	Deep Shelf	Nearshore	Deep Fjord
**Different taxonomic level**						
Genus level analysis	119	5.89	1.13	2.85	0.91	0.99
Family level analysis	90	9.92	3.12	4.89	0.86	1.05
Genus level analysis (with excluded records)	119	7.69	1.91	3.95	0.75	1.08
Family level analysis (with excluded records)	90	12.04	3.41	6.67	0.81	1.16
**Specific zooplankton groups**						
Holoplankton	125	6.61	0.24	2.50	3.71	0.16
Meroplankton	35	53.99	13.95	21.98	13.40	4.66
Crustacean holoplankton	84	11.18	0.56	5.19	5.35	0.08
Non-crustacean holoplankton	41	40.28	6.29	18.87	6.96	8.17
Copepods	59	17.20	1.83	8.04	6.58	0.75
Holoplankton without copepods	66	16.34	2.90	7.28	4.68	1.48
**Including species present in < 3 % of samples**						
All 455 species	455	0.24	0.03	0.13	0.0	0.08
Holoplankton only (394 species)	394	6.66	0.22	2.55	3.71	0.19
Meroplankton	61	53.73	13.86	21.84	13.26	4.77
Crustacean holoplankton	283	10.86	0.54	4.78	5.46	0.08
Non-crustacean holoplankton	111	40.20	6.24	18.84	6.93	8.20
Copepods	172	17.52	1.59	8.14	7.04	0.75
Holoplankton without copepods	227	16.29	2.85	7.28	4.68	1.48
**Different community data type**						
Presence-absence	160	9.78	2.50	4.25	1.85	1.18
Biomass	160	13.28	2.28	5.48	4.70	0.81

## Conclusions

Selecting the appropriate method for bioregionalization is data specific and in this study, we outlined a process to systematically identify a data-driven approach for bioregionalization. After comparing nine cluster analysis approaches using ten goodness of clustering indices, we selected the K-means clustering of the log-chord transformed abundance as the optimal method for identifying bioregions. Although the dataset was curated for consistency, it still contained heterogeneity on how zooplankton were collected in a 20-year period by different research programs. We conducted robustness tests by comparing the bioregionalization of the overall data to the results of various subsets of the data. Throughout the robustness tests, the highest uncertainty was accounted for by the Deep Shelf bioregion. We hypothesize that this is because of it being a transition between oceanic and neritic environments. All in all, the robustness analysis demonstrated that the bioregionalization was consistent to random variations in the data and the sampling method heterogeneity. Unsurprisingly, the bioregionalization was sensitive to the temporal and taxonomic extent of the zooplankton data as this limits the inclusion of many indicative species that periodically appear in the zooplankton net samples. Nonetheless, the bioregionalization was robust to the frequency of sampling in a year and between years, the spatial resolution of sampling, the taxonomic specificity in identify species, and limiting the analysis to relatively more commonly observed species. In validating the zooplankton bioregionalization, we present in the companion paper that the bioregions significantly differ in physical and biological oceanographic characteristics and zooplankton community composition [12]. Zooplankton community data often include many species and for our relatively large dataset, the log-chord transformed abundance allowed us to directly apply the K-means cluster analysis which is advantageous since the same transformed species abundance data can be used in other complementary multivariate analyses.

## CRediT authorship contribution statement

**Patrick R. Pata:** Conceptualization, Methodology, Software, Formal analysis, Data curation, Writing – original draft, Writing – review & editing, Visualization. **Moira Galbraith:** Conceptualization, Investigation, Resources, Data curation, Writing – review & editing. **Kelly Young:** Conceptualization, Investigation, Resources, Data curation, Writing – review & editing. **Andrew R. Margolin:** Conceptualization, Investigation, Software, Data curation, Writing – review & editing. **R. Ian Perry:** Conceptualization, Resources, Writing – review & editing, Supervision. **Brian P.V. Hunt:** Conceptualization, Methodology, Formal analysis, Writing – review & editing, Supervision, Funding acquisition.

## Declaration of competing interest

The authors declare that they have no known competing financial interests or personal relationships that could have appeared to influence the work reported in this paper.

## Data Availability

The zooplankton data were sourced from the Fisheries and Oceans Canada, Institute of Ocean Science Zooplankton Database and is accessible online.
